# Supplementary material for: ER exit sites mediated by the COPII adaptor sec24D selectively recruit lipid raft-preferring proteins for rapid ER export
Source: Nat Commun. 2025 Nov 27;16:10694. doi: 10.1038/s41467-025-65726-8 (PMC12660933; doi:10.1038/s41467-025-65726-8)
Supplement: Supplementary file 1 — Supplementary Information [file 41467_2025_65726_MOESM1_ESM.pdf]

Supplementary Information for

ER exit sites mediated by COPII adaptor sec24D selectively recruit lipid raft-preferring proteins for rapid ER export

Ivan Castello-Serrano[1,2*], Shikha Dagar[1,3], Rossana Ippolito[1,2], Kandice R. Levental[1*], Ilya Levental[1*]

[1]Department of Molecular Physiology and Biological Physics, Center for Membrane and Cell Physiology, University of Virginia, Charlottesville, VA 22904
[2]Current address: IRCCS Ospedale San Raffaele, Via Olgettina 60, Milan 20132
[3]Current address: Department of Microbiology and Immunology, University of Illinois Chicago, Chicago, IL 60607
[*]Corresponding authors: castelloserrano.ivan@hsr.it, krl6c@virginia.edu, il2sy@virginia.edu

This file contains 8 Supplementary Figures and associated figure legends.

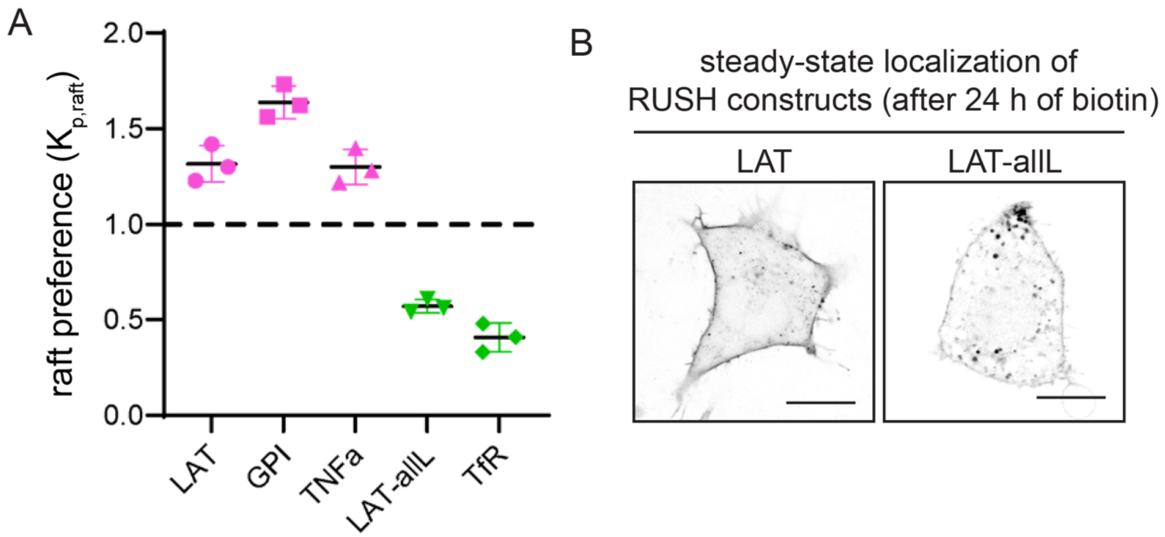

Supplementary Figure 1. Raft phase partition coefficients (Kp) for several full-length proteins of interest in this study calculated from GPMV images. (A) Values >1 represent proteins enriched in the raft-like ordered phase of Giant Plasma Membrane Vesicles, whereas <1 represents enrichment in the disordered, non-raft phase. Symbols represent means of independent experiments with >10 vesicles in each. (B) Representative confocal images showing steady-state localization of RUSH LAT and LAT-allL constructs, i.e. 24 h after biotin introduction. Scale bars = 5 µm.

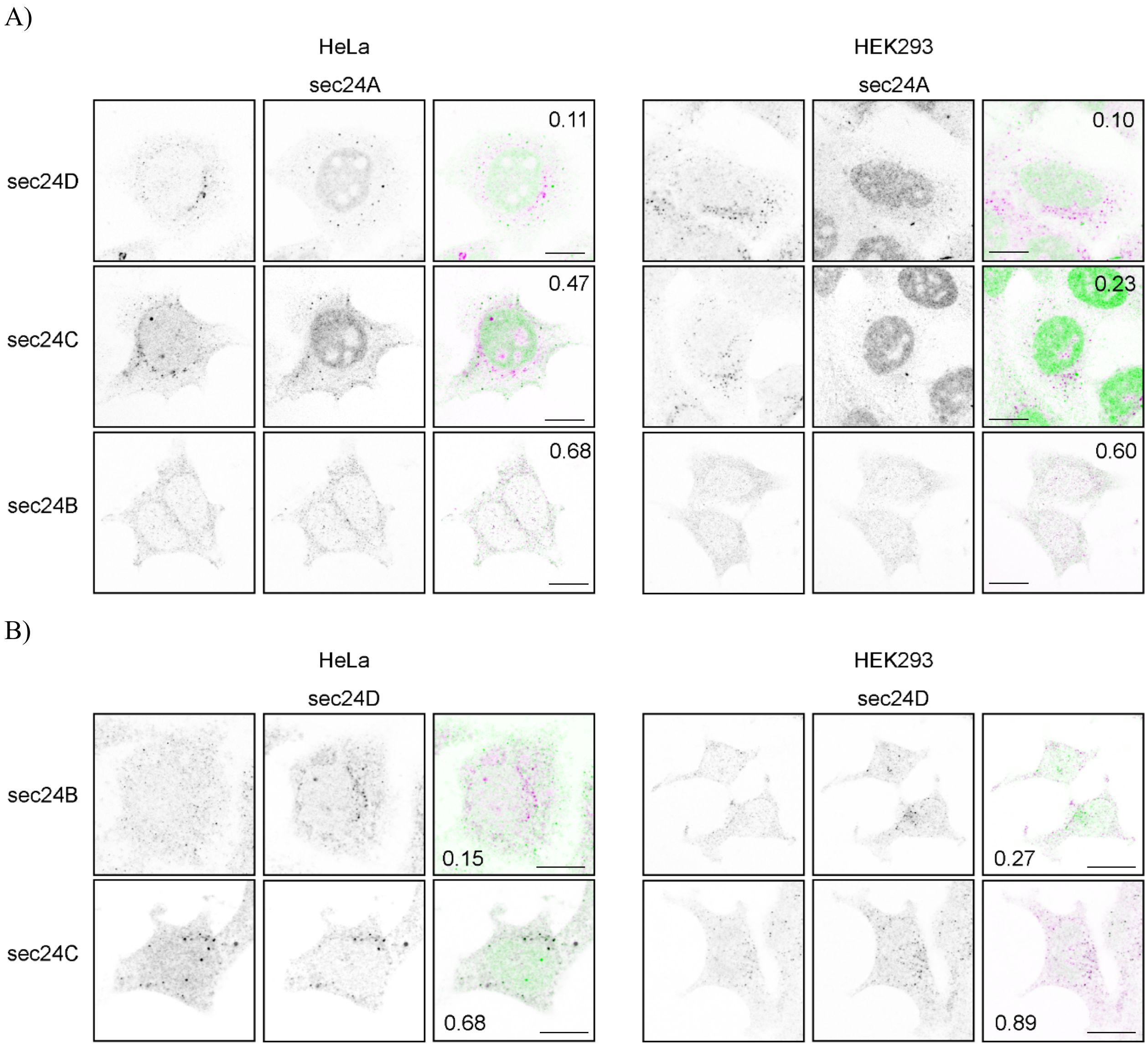

Supplementary Figure 2. Immunofluorescence showing colocalization of the four sec24 isoforms in two cell lines. (A) sec24A localizes to cytoplasmic puncta which are often coincident with puncta of sec24B, but not sec24D, in both HeLa and HEK293. sec24C is somewhat less clear, with exclusion from sec24A in HEK293, but moderate colocalization in HeLa. (B) Sec24D patterns follow the same trend in both cell lines, with high colocalization with sec24C but not sec24B. Values represent average colocalizations from >10 cells. Scale bars = 5 µm.

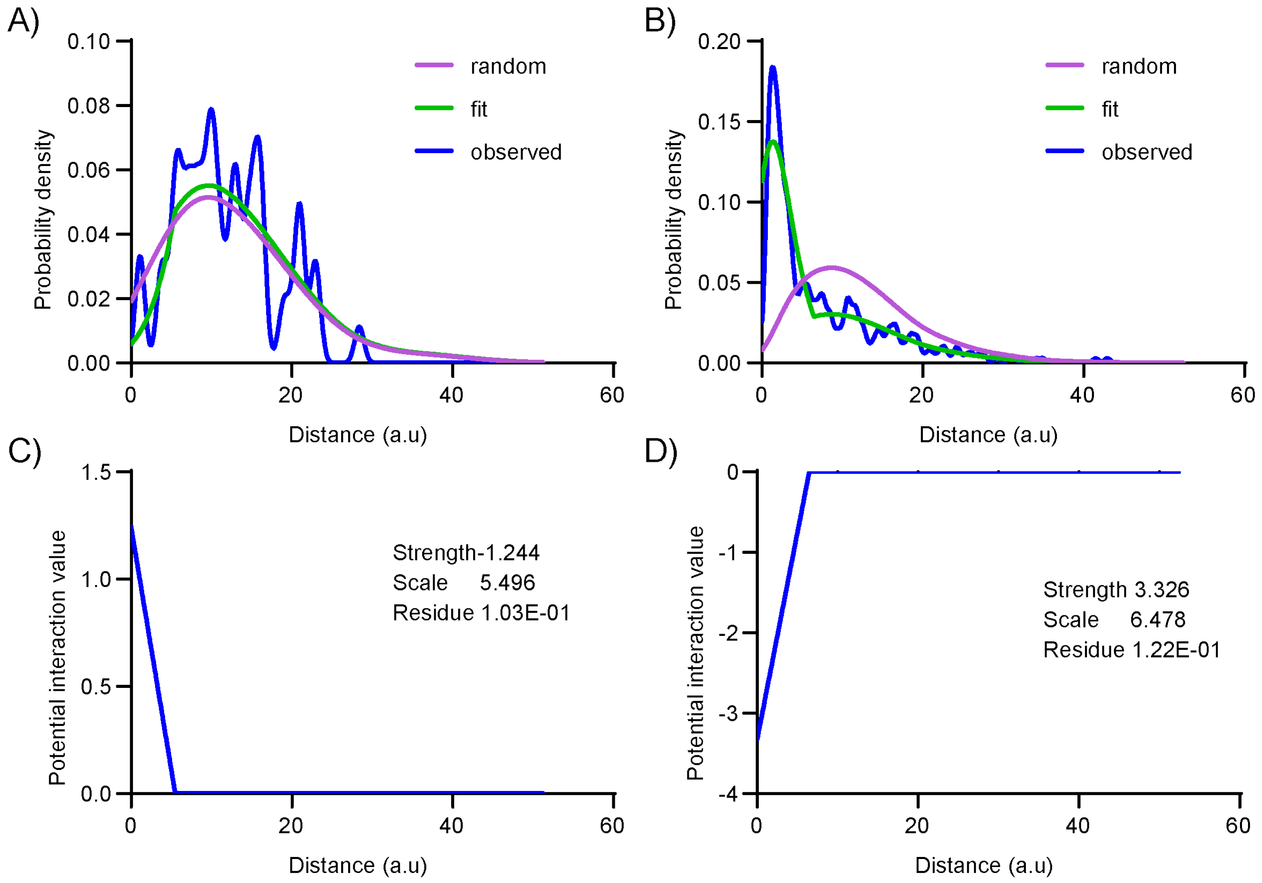

Supplementary Figure 3. Throughput for the Mosaic analysis for spatial pattern and interaction. (A) Probability of sec24A-positive pixels as a function of distance from Golgi (i.e. 58K-positive) pixels. The blue curve shows the observed correlations from the images, green is the fit of the observed data to the Plummer potential model, and purple shows the expectation for an uncorrelated (i.e. random) distribution between sec24A and Golgi. These curves suggest that Sec24A is not correlated with the Golgi template, with the fit overlapping well with random distribution. (B) In contrast, sec24D is positively correlated with Golgi staining, showing a much higher-than-random likelihood of nearby staining. (C) These data can be used to infer an 'interaction potential' between sec24 isoforms and the Golgi marker. Positive values of 'interaction strength' (and negative values of 'potential interaction value') reveal attractive interactions between the components, which is the case for sec24D and Golgi, but not sec24A.

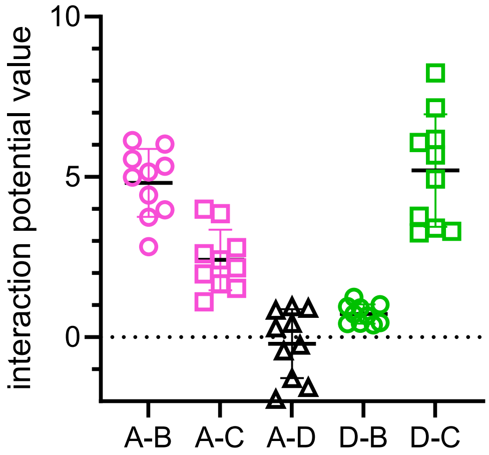

Supplementary Figure 4. Interaction potential of endogenous sec24 isoforms from immunostaining. Positive interaction potential reveals attractive interactions between components (see Supp Fig 3). sec24A&B and sec24C&D are strongly correlated, whereas all other combinations are weakly (or not at all) interacting.

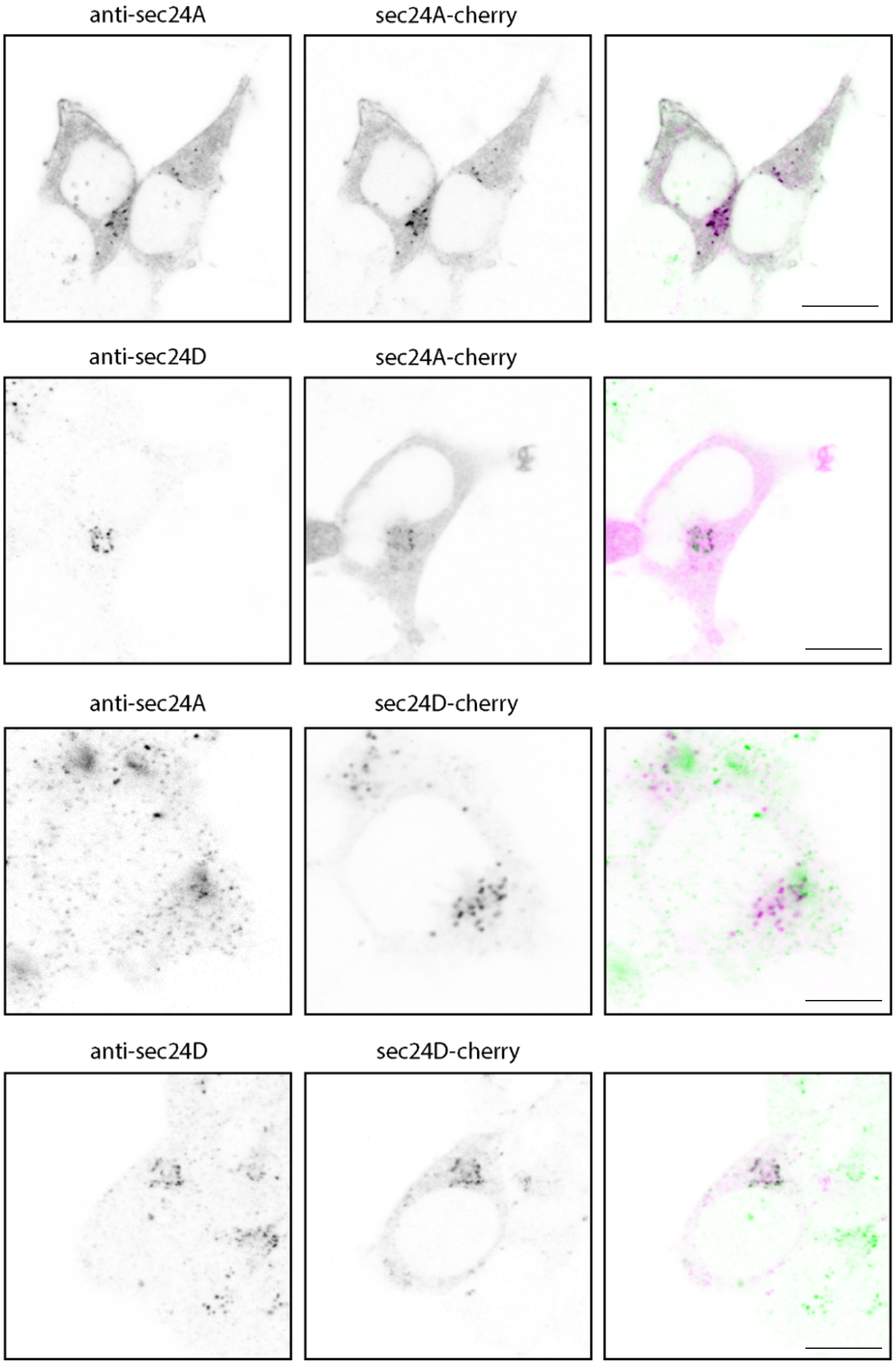

Supplementary Figure 5. Confirming the specificity of anti-sec24 antibodies in cells transfected with sec24A/D. Representative images of cells transfected with sec24 isoforms fused to fluorescent proteins and subsequent staining with antibodies against either the same isoform or the "opposite" one. Staining shows that the antibodies can distinguish the various isoforms. Black in the merge channel represents colocalization. Scale bars = 5 μm.

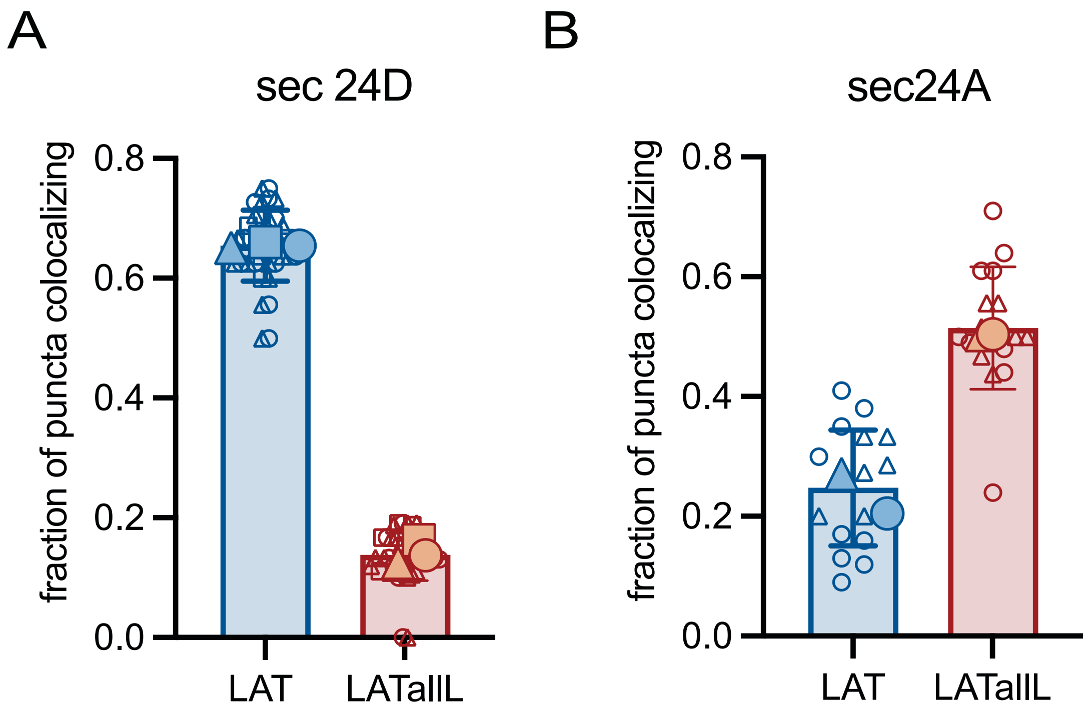

Supplementary Figure 6. sec24 isoforms colocalize with cargo proteins based on their raft affinity. Quantification of LAT or LAT-allL puncta observable 10 minutes of biotin treatment to release ER RUSH with puncta of either (A) sec24D or (B) sec24A. Empty symbols correspond to individual cells, solid symbols to means of 3 individual experiments with >10 cells each.

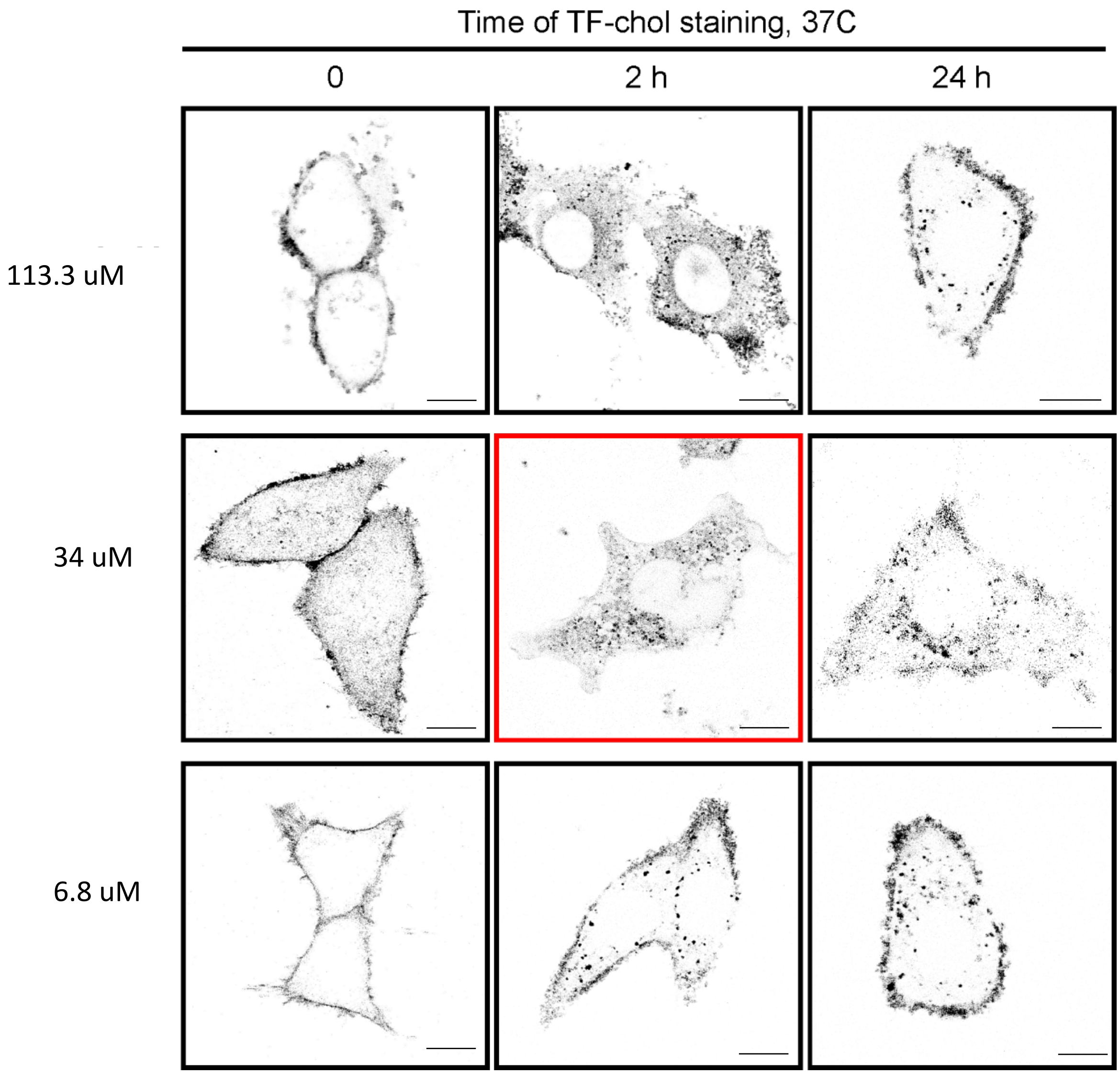

Supplementary Figure 7. TF-chol staining is dependent on time and labeling concentration. Representative images of several conditions to observe the cellular structures stained with TF-chol as a function of labeling concentration and time. The red-framed image indicates the conditions (34 µM, 2 hour chase at 37°C) used to observe the ER localized puncta. Scale bars = 5 µm.

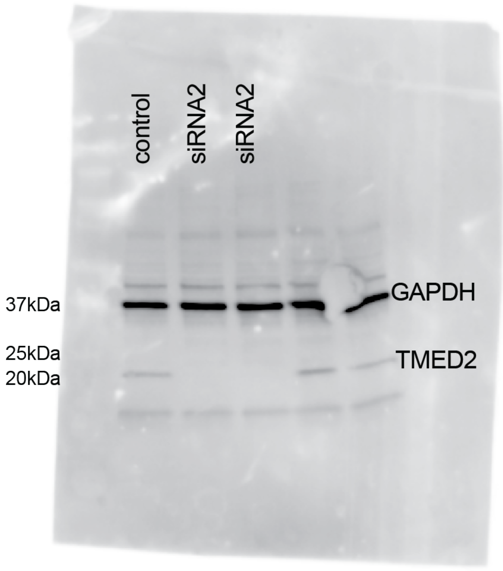

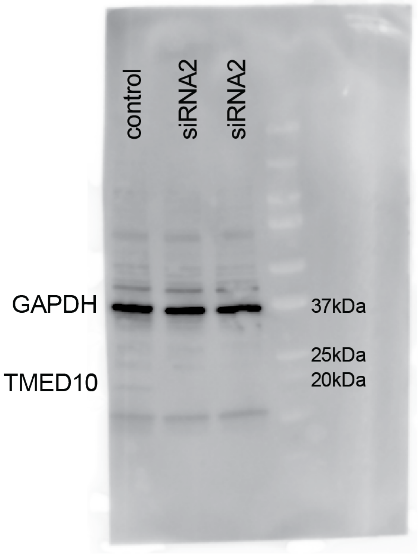

Supplementary Figure 8. Full, uncropped Western blot of TMED2, TMED10, and GAPDH from figure 4.
